# Molecular Labelling Tool for Cereal Genetic Resources Management Derived from Barley and Tetraploid Wheat Genebank-Genomics Projects

**DOI:** 10.3390/plants15081219

**Published:** 2026-04-16

**Authors:** Workie Zegeye, Amanda Burridge, Ajay Siluveru, Simon Orford, Liz Sayers, Richard Goram, Richard Horler, Gary Barker, Noam Chayut

**Affiliations:** 1 Germplasm Resources Unit, John Innes Centre, Norwich Research Park, Colney Lane, Norwich NR4 7UH, UK; workie.zegeye@jic.ac.uk (W.Z.); amanda.burridge@bristol.ac.uk (A.B.); ajay.siluveru@jic.ac.uk (A.S.); simon.orford@jic.ac.uk (S.O.); richard.goram@jic.ac.uk (R.G.); richard.horler@nbi.ac.uk (R.H.); 2 Department of Agricultural Biotechnology, University of Gondar, Gondar P.O. Box 196, Ethiopia; 3 School of Biological Sciences, University of Bristol, 24 Tyndall Avenue, Bristol BS8 1TQ, UK

**Keywords:** DNA fingerprint, DNA barcoding, genebank, KASP marker, minimal marker set

## Abstract

Globally, 5.94 million accessions are conserved across 867 genebanks, of which 41.5% (2.47 million) are cereal crop accessions. Only a small portion of global germplasm diversity has been marker-genotyped or genome-sequenced. Accurate identification of genebank accessions is essential to improve the efficiency and effectiveness of global genebanking. It is crucial for preserving the legacy knowledge associated with the germplasm and for maintaining its value to current plant science and breeding efforts. Existing practices generally fall into two categories: either expensive and complex, or inefficient, labour-intensive, and inaccurate. The first relies on high-resolution genomic sequences or saturated markers, while the second relies on morphological comparisons of regenerated plants with historical records. We propose a genotyping method based on a minimal set of Single Nucleotide Polymorphism (SNP) markers and exemplify its use on a genebank scale. We identified a small, effective set of SNPs that can differentiate between the global diversity of genebank accessions of barley (*Hordeum vulgare* and *Hordeum spontaneum*) and tetraploid wheat collections (*Triticum turgidum)* maintained at the Germplasm Resources National Capability at the John Innes Centre, UK. This approach offers a straightforward, automatable, and inexpensive alternative to traditional genebank crop descriptors used during seed regeneration and distribution. By establishing the minimal genomic resolution needed to distinguish genetically distinct accessions, we show that as few as 24 and 25 carefully chosen SNP markers for barley and durum wheat, respectively, can effectively differentiate individual accessions. Unlike morphology-based identification, which can detect mislabelling or contamination but often cannot prevent or correct such errors, our SNP-based molecular labelling enables error correction and the retrieval of lost germplasm identity. This study highlights how accuracy and reliability in germplasm management can be improved without costly whole-genome sequencing or resource-intensive analysis. We discuss the impact of this method on enhancing quality assurance in genebanks and its broader usefulness for the user community.

## 1. Introduction

The systematic global ex situ conservation of Plant Genetic Resources (PGRs) in genebanks (also called seedbanks) started some 50 years ago, following the recognition that the breadth of crop gene-pool diversity is threatened in their agro-bio environment [1,2,3]. The State of the World’s Plant Genetic Resources for Food and Agriculture report lists 850 national, 13 international, and four regional genebanks that collectively conserve 5.94 million accessions. Of these, 2.47 million (41.5%) are cereal seed samples [1].

Barley (*Hordeum vulgare*) and durum wheat (*Triticum turgidum* subsp. *durum*) are the fourth and tenth most globally cultivated cereal crops, with annual worldwide production of approximately 146 and 35–40 million tonnes, respectively [2]. Barley is cultivated in at least 103 countries, and durum wheat is a staple crop in several regions of the Near East, North Africa, Europe, and Asia [3]. Barley and durum wheat are cultivated on 46.3 and 18 million hectares worldwide, producing grains valued at 31.3 and 18.2 billion USD, respectively [4]. The agricultural importance of the two crops is reflected in extensive global ex situ conservation efforts. Barley and wild relatives (i.e., genus *Hordeum* L.) are present in at least 129 institutions worldwide, collectively maintaining about 400,000 accessions [5]. Extensive collections of durum wheat are maintained by several international genebanks, such as CIMMYT (~19,000 accessions [6]), the Australian Grains Genebank (~11,000 accessions [7]) and national European genebanks (~17,000 accessions listed in EURISCO [8]).

The conservation of wild and cultivated PGRs is essential to long-term food security and for addressing the multifaceted challenges facing agriculture in the context of climate change, habitat loss, and the decline of agro-biodiversity [1]. However, maintaining a large number of accessions presents significant challenges, particularly in ensuring accurate identification and separation for maintaining the integrity of conserved materials and their associated data [9,10,11].

Ex situ conserved seed shelf life can be prolonged for decades, but not in perpetuity. Thus, seed lots of old age or low-quality samples, as well as those distributed to genebank users, need to be regenerated periodically. While the regeneration cycles are necessary, they can negatively influence the PGR value primarily due to operational errors related to the identity of an accession (e.g., mislabelling or seed mix-ups during sowing or harvest), to unintended selection, and to genetic drift [12,13,14]. Uncertain seed lot identity disassociates the germplasm accessions from their related background data. Therefore, while the biological genetic diversity itself is not literally lost, the PGRs become much less usable for crop science and breeding.

Accurate identification of PGR accessions is critical to enhance the global efficiency and effectiveness of genebanking and prevent the decay of collection value. It is key to preserving the legacy knowledge and its potential future utilisation. Current common practices can be largely divided into two groups: those that are expensive and complex, and those that are inefficient, labour-intensive, and often inaccurate. The first group relies on high-resolution genomic sequencing or a saturated marker set (also called genebank-genomics), and the latter on morphological comparisons of regenerated plants with historical records. Some recent examples of cereal genebank-genomic projects include the DArTseq^TM^ genotyping of ~80,000 wheat accessions in the CIMMYT genebank [6], the Genotype by Sequencing (GBS) of ~22,600 barley accessions [15], and short-read Illumina sequencing of global wheat [16], rice [17,18], and maize [19,20] panels. This breadth of high-resolution cereal genebank-genomic data has not yet been exploited for practical germplasm labelling, which requires selecting the fewest possible markers to label the broadest possible conserved diversity. Morphological approaches for PGR identification and characterisation are often described as part of crop conservation strategies, many of which were recently standardised by a considerable community effort, centralised, and published by the Global Crop Diversity Trust [2].

The phenotyping descriptors are often highly valuable for genebank users [21], and can be used to detect operational errors following a regeneration cycle, but are often not suitable for resolving the true identity after mislabelling and seed mix-ups are detected. On the other hand, genebank-genomics data is restrictive: most genebank accessions lack genomic data, and most genebank curators lack the bioinformatic resources needed for its interpretation for PGR accession identification.

Genomic barcoding is a promising tool for effectively managing plant genetic resources [22,23]. While genomic barcoding offers a powerful approach, doing so by sequencing large germplasm collections is often prohibitively expensive. Additionally, the complexity of full-sequence or high-density marker data makes it impractical for routine quality control and quality assurance during regeneration and distribution, respectively [24].

To address these limitations, we identify and demonstrate a low-cost, simple, reliable, and rapid genotyping approach: genomic barcoding using a minimal, optimised set of Single Nucleotide Polymorphism (SNP) markers. While marker technology is widely used, this study offers a novel application. Here, the Minimal Marker Sets (MMSs) are specifically designed for routine genebank operations. Unlike previous low-density SNP panels and SSR assays, which have mainly been used to support breeding (marker-trait associations) and population genetics studies, these MMS panels are, to the best of our knowledge, the first example of cereal genebank molecular barcoding, designed to manage cereal ex situ plant genetic resources. They were specifically chosen to optimise their capacity to verify seed identity, ensure quality control during regeneration, detect admixture or contamination, and track samples over time. The MMS applicability was validated across the full range of conserved diversity, including crop wild relatives, traditional landraces, and modern cultivars from diverse geographic origins. This unusually broad scope is adequate specifically for genebank needs and is different from common marker optimisation studies, which often focus on a specific diversity cross-section, usually of elite germplasm or a single origin. Overall, these MMSs are not just simplified marker panels; they are practical genomic tools designed to optimise cereal conservation delivery. Importantly, they bridge the gap between high-resolution genomics delivered by academia and the practical, cost-effective needs required by conservationists for large-scale genebank management.

Here, we develop and test sets of 25 and 24 highly informative KASP markers, as a practical method for molecular barcoding of tetraploid wheat and barley (*Hordeum vulgare* and *Hordeum spontaneum*), respectively. This new approach provides an affordable, scalable, and efficient solution for genebank management, ensuring accurate identification while optimising resources [25].

## 2. Results

To develop the MMSs, we first defined the minimal requirements for uniquely identifying a genebank accession, which would determine the resulting genomic resolution and separation power. For durum wheat, we aimed to distinguish between the 345 durum accessions of the A.E Watkins Wheat Landrace Collection (Supplementary Table S1), a century-old, UK-curated, largely untapped collection from 25 countries. For barley, we aimed to distinguish between 500 randomly selected accessions from the Leibniz Institute of Plant Genetics and Crop Plant Research (IPK) collection (Supplementary Table S2). These are defined as the MMS training populations and are distinct from the analysis populations used for the MMS validation (Table 1).

### 2.1. Barley

**Figure 1:**
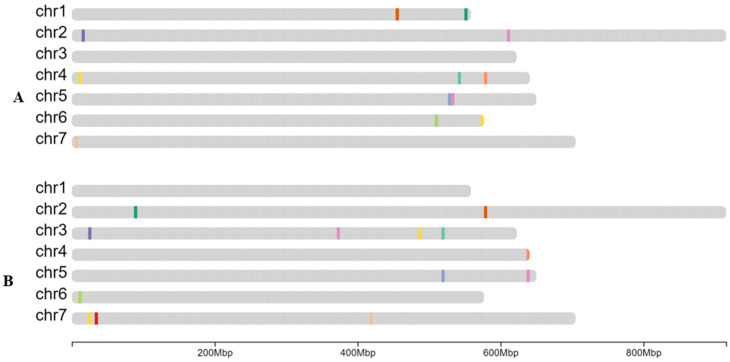
Distribution of the 25-BMMS. (**A**). The initial 12 markers throughout the barley genome. (**B**). The distribution of 13 additional markers throughout the barley genome. Each coloured line represents one marker. The mega base pair (Mbp) scale is shown at the bottom.

For barley, we used previously published genebank-genomic resources consisting of 171,263 SNPs across 22,626 genebank accessions [12]. We filtered the dataset down to 10,698 highly informative SNPs and the accession number down to ~7000 highly homozygous lines. The filtered SNPs had >95% call rate and >0.05 (5%) minor allele frequency (MAF). The retained wild and domesticated barley accessions had a high call rate (>99.5%) and low heterozygosity (<0.1%). From this set, a random sample of 500 varieties and the 10,689 SNP rows passing filters was run through the minimal marker program This generated 12 SNPs able to discriminate 99.9% of the accessions, prompting us to use them as our Barley Minimal Marker Set (BMMS) starting point, as illustrated in Figure 1A and Supplementary Table S2. As an additional 16 SNPs were required to achieve 100% discrimination in silico, the decision was made to first evaluate the 12 SNP BMMS

The 12 BMMS were first evaluated for their ability to distinguish 96 barley accessions from the BBSRC Small-Grain Collection housed in the Germplasm Resources Unit (GRU) (Supplementary Table S3). A unique marker combination was identified for 90 out of the 96 targeted lines, while six lines could not be uniquely distinguished (a discrimination power of 93.75%) (Table 1). Applying the same 12-BBMS to 384 barley landrace accessions (Supplementary Table S3) resulted in a discrimination power of only 70.57% (Table 1). So, while the markers successfully discriminated 99.9% of the training population (in silico), their wet lab performance on the GRU barley landrace set was suboptimal. To improve resolution, we refined the algorithm to incorporate existing markers, as described in the Materials and Methods. Adding 13 markers identified in the first or second iterations of the algorithm resulted in a 25-marker BMMS (Figure 1B).

We then re-analysed the same accessions (96 UK-listed advanced barley cultivars and 384 landraces from 49 countries) using the expanded 25-marker set. With the exception of one pair of accessions, all the cultivars (94 out of 96) were assigned a unique marker combination identifier (97.92% separation), and the landrace discrimination power increased from 70.57% to 90.36% (Table 1). Among the 25 markers, one was consistently monomorphic across all the 480 tested accessions and was thus excluded, resulting in the 24BMMS (24 Barley Minimal Marker Set) (Supplementary Table S4). We continued to validate the 24BMMS by genotyping an additional 284 landraces from 31 countries, totalling 668 landraces (Supplementary Table S3), achieving a 92.22% discrimination power.

Approximately 8% of the 668 barley landrace accessions genotyped using the 24BMMS (a total of 52) were unexpectedly not identified as unique (Supplementary Table S5). We reviewed the passport data of these accessions available on https://www.seedstor.ac.uk. More than half (27 out of 52) (Supplementary Table S5) were found to share the same pedigree or have a similar source (e.g., donated from the same country at the same time by the same donor). These accessions, which were presumably considered unique by the donors, fall below the 24BMMS discrimination capacity. For the remaining 3.7% (25 out of 668) (Supplementary Table S5) accessions that were not distinguished, no explanation can be found in their passport data. These may represent duplicated accessions (genebank errors) or distinct accessions that fall below the identification tool resolution. In summary, the 24BMMS tool assigned a unique marker identifier to 92.44% of the listed cultivars and landrace genebank accessions from distinct genetic backgrounds.

We also investigated whether the 24BMMS could be used to define the genetically unique identity of wild barley (*Hordeum spontaneum*). As expected, given the close relatedness of the species [26,27], the markers were successfully amplified in *H. spontaneum* and exhibited sufficient levels of polymorphism in both species (Supplementary Table S6). We hypothesised that the 24BMMS would distinguish between distinct wild barley populations but would not capture subtle genetic variations within an individual wild population. In other words, the molecular barcoding resolution was expected to reflect the accepted definition of a “wild germplasm genebank accession,” which aims to conserve the intrinsic diversity of a wild population [28].

**Figure 2:**
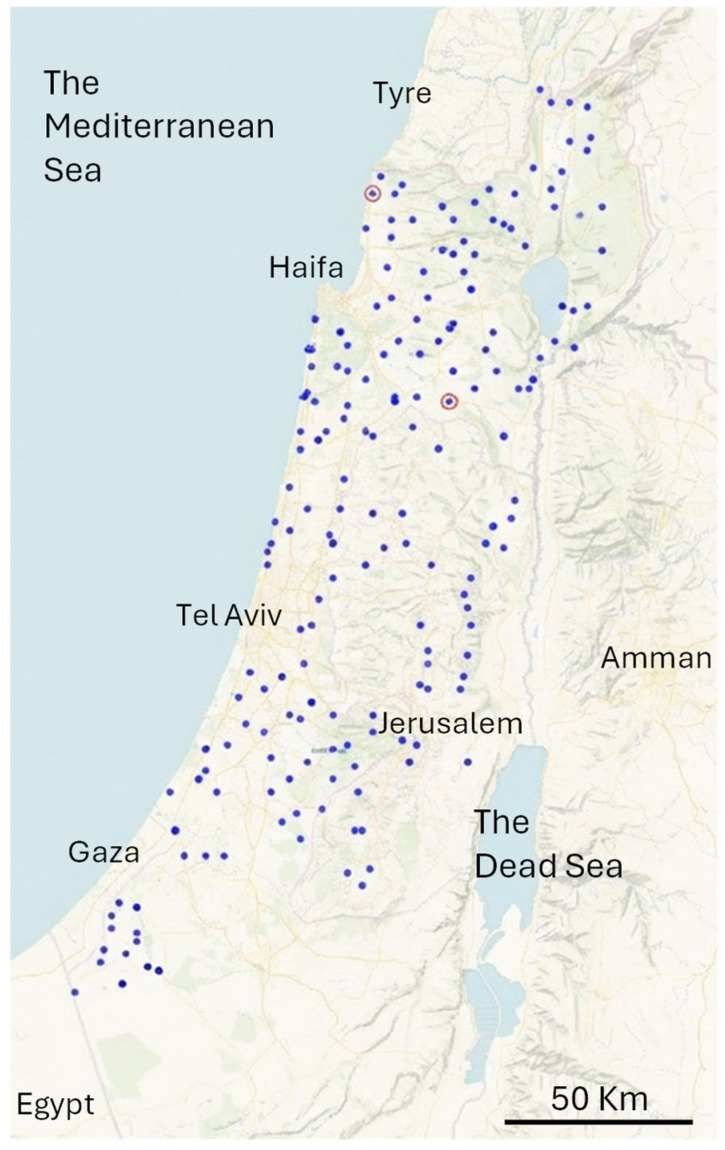
Geographic distribution of wild barley collection sites on a physical map of Israel/Palestine and the surrounding territories; green background colour indicates plains of low-lying land, yellow/brown shows higher elevation and mountains, and blue represents water bodies. Each blue dot depicts the location of a collection site for a wild population, separated from all others by minimal marker-set genotyping. The two red circles denote the locations from which the two unseparated samples were obtained. A 50 Kilometre scale bar is added at the bottom-right corner of the figure.

To test this, we selected 95 seeds collected from 95 distinct geographic locations within the native range of *H. vulgare* across Israel/Palestine and surrounding territories (Figure 2). We expected a high level of genetic differentiation among these populations. Indeed, the 24BMMS successfully distinguished between 97.89% of the sampled wild populations (Table 1). We then examined the remaining two accessions (2.11%) that could not be separated to determine whether they came from nearby populations. As shown in Figure 2, these populations (indicated by red circles) were not located near each other, suggesting that their genetic similarity is not due to geographic proximity.

To further test the hypothesis that the resolution power of the 24BMMS would be lower when attempting to distinguish between individual lines within a wild barley population, we extracted DNA from four to ten plantlets, representing the progeny of different plants sampled in 16 defined geographic locations. The 24BMMS discriminated 50% to 100% of individuals across the 16 tested populations (*n* = 98), with an average of 92.76% of progenies identifiable through unique marker combinations (Supplementary Table S7).

For cultivated barley, the 24BMMS was designed to differentiate between genebank accessions, such as landraces and listed cultivars that have evolved and adapted in diverse agro-bio-geographic environments and therefore harbour a distinct genetic background and unique genomic fingerprints. We hypothesised that the genomic resolution of the 24BMMS would not necessarily be sufficient to identify differences between breeder lines, such as single-seed descent, lines derived from the same landrace population, or from mapping populations with only two, often closely related, parental cultivars. To evaluate the effectiveness and quantify the limitations of the 24BMMS, we assembled a panel of 96 accessions grouped into 13 related breeder lines, with 4–9 accessions in each group (Supplementary Table S8). The 24BMMS was able to discriminate 88.54% of the breeder line groups and an average of 88.14% within the breeder lines. Expectedly, the breeder line separation resolution was lower than that observed for landraces and cultivars.

### 2.2. Durum Wheat

**Figure 3:**
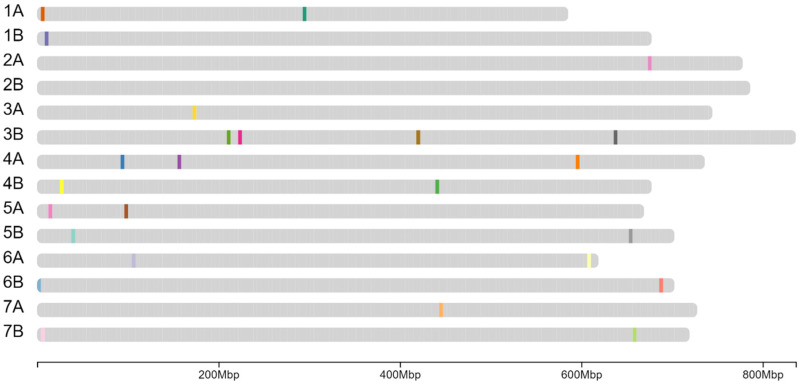
Distribution of the 25DMMS throughout the durum wheat genome. Each coloured line represents one marker. The mega base pair (Mbp) scale is shown at the bottom.

For durum wheat, we genotyped the 345 Watkins landrace accessions using the 35K Axiom Wheat Breeders’ array and employed the same algorithm previously applied in barley to design a Durum Minimal Marker Set (DMMS). The minimal SNP discovery analysis performed on the 345 Watkins landraces yielded 21 markers capable of discriminating all 345 accessions in silico (Supplementary Table S1). As this genotyping array has been updated to the TaNGv1.1 Axiom array [29], we repeated the analysis using the 7144 SNPs common to both the 35K Wheat Breeders’ array and TaNGv1.1. This reduced input set identified 27 markers required to resolve all durum lines. To ensure compatibility and reliability, these markers were further filtered for clear array separation, low heterozygosity, a call rate greater than 95%, and an MAF above 5%. This filtering process resulted in the final 25-marker DMMS (25DMMS) panel (Figure 3 and Supplementary Table S4).

We subsequently validated the 25DMMS for practical molecular identification of durum wheat (*T. turgidum* ssp. *durum*). Wet lab application of the 25DMMS to the Watkins landrace training population achieved a 98.84% discrimination efficiency (Table 1). We further validated the 25DMMS on 160 conserved landraces and modern durum cultivars, available in the GRU as part of the BBSRC Small-Grain Cereal Collection (Table 1 and Supplementary Table S9). These accessions were introduced to the Plant Breeding Institute (Cambridge) between 1962 and 1989 from 24 countries. Within this highly diverse set, the 25DMMS separated 97.5% of the accessions (Table 1), confirming the tool’s reliability for genebank management purposes. The remaining 2.5% (4 out of 160 accessions, highlighted in yellow in Supplementary Table S9) were found to share the same pedigree or have a similar source (donated from the same country at the same time by the same donor). These accessions, which were presumably considered unique by these donors, fall below the 25DMMS separation capacity.

To assess the global applicability of the 25DMMS, we applied it to a representative panel of 260 durum wheat accessions sourced from ICARDA (n = 168), the Australian Grain Genebank (AGG) (n = 48) and the United States Department of Agriculture (USDA) (n = 44), forming part of the previously assembled Global Durum Panel [30] (Supplementary Table S10). In this diverse global context, the panel discriminated 97.31% of accessions (Table 1 and Supplementary Table S10), underscoring its robustness and efficiency for accurate genetic discrimination across a wide range of domesticated *Triticum durum* germplasm.

We further investigated whether the 25DMMS fingerprinting approach could be used to define genetically unique identities of tetraploid wheat wild relatives. We therefore genotyped 122 *Triticum turgidum* accessions belonging to the subspecies *carthlicum*, *dicoccoides*, *dicoccum*, *polonicum*, *turanicum*, and *turgidum*; these were sourced from the three aforementioned genebanks, and form part of the previously assembled Tetraploid Wheat Germplasm Collection [31]. As expected, given the close relatedness of the species [27], all markers were successfully amplified, exhibiting high levels of polymorphism across the six wild subspecies groups and successfully discriminating 89.44% of the studied accessions (Table 1 and Supplementary Table S11).

**Figure 4:**
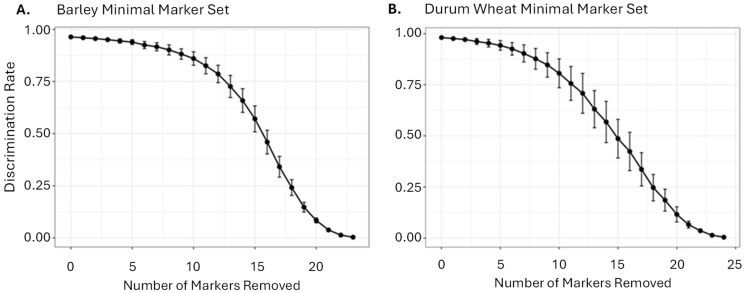
Robustness of the 24 Barley Minimal Marker Set (24BMMS) (**A**) and 25 Durum Wheat Minimal Marker Set (25DMMS) (**B**) under simulated marker failure. The original marker set achieved a high discrimination rate. As markers were progressively removed, the discrimination rate declined gradually. Markers were removed at random subsets ranging from 1 to n − 1, and 200 independent simulations were run for each scenario. The average discrimination power (black dot) and standard deviation (error bars) are depicted.

Lastly, we evaluated the core polymorphism statistics of the 24BMMS and 25DMMS across the different germplasm population groups (Supplementary Tables S12 and S13) and simulated their failure scenarios (Figure 4) to quantify the theoretical rigour underpinning the aforementioned empirical validation results. The wet lab validation MAF results for 24BMMS and 25DMSS ranged from 0.198 to 0.496 and from 0.024 to 0.496 and averaged 0.381 and 0.225, respectively. The Polymorphic Information Content (PIC) for 24BMMS and 25DMSS ranged from 0.267 to 0.375 and from 0.046 to 0.375 and averaged 0.353 and 0.246.

In the simulated failure scenarios, the original MMS achieved the expected high discrimination rate. As a growing number of random markers were progressively removed, the discrimination rate gradually declined. However, even after removing up to five randomly selected markers (simulating an extremely high level of wet lab failure), the MMS retained a high level of discriminatory power: 93.91% for barley and 94.24% for durum wheat (Figure 4).

In summary, the MMS developed in this study achieved a high level of accession discrimination across highly diverse global collections of domesticated and wild barley and tetraploid wheat, delivering a novel and practical DNA fingerprinting tool for cereal genebank management.

## 3. Discussion

### 3.1. From Genebank-Genomics to Practical Germplasm Management Tools

The conservation and management of publicly available Plant Genetic Resources (PGRs) are fundamental to ensuring global food security. More than 850 genebanks serve globally as repositories of over 5.9 million PGR accessions for breeding and research applications [1]. Maintaining large PGR collections presents significant challenges, particularly in ensuring accurate identification, minimising redundancy, and effectively ensuring the genetic integrity of collections and the genetic purity of lines. Decades of conservation necessitate periodic seed regeneration. Inevitable human errors during the regeneration of large collections (e.g., seed mix-ups, field mislabelling, mistakes during sowing or harvesting) and unintentional selection (e.g., by keeping more seeds of high-yielding individual plants) compromise the value of the conserved PGRs.

The GRU is the UK’s public genebank for small-grain cereals and grain legumes, focusing on promoting FAIR (Findable, Accessible, Interoperable, and Reproducible) germplasm and data resources to advance crop science and breeding [32]. Generating high-resolution genomic data to characterise and study a broad germplasm diversity set is often termed “genebank-genomics”. Several of the GRU [16,33,34] collections were recently (or are currently) subjected to such high-resolution Genebank-Genomic projects [13,30,31], blurring the boundaries between crops and model plants [35], thus bringing about a step change in PGR usability for plant science and genomic-assisted breeding. However, these high-resolution datasets do not yield a ready-to-use genomic tool for the practical management of genebank accessions due to the complexity of the resulting data. Whole-genome sequences or high-density marker data are impractical for routine germplasm quality control and quality assurance during regeneration and distribution. Here, we use existing genebank-genomic data generated in the GRU and in IPK [15] to develop and test a frugal approach to molecular labelling using a minimal set of highly informative KASP markers.

This provides an affordable, scalable, and efficient solution for genebank management, ensuring accurate identification and resource optimisation. The method requires access to a basic molecular laboratory and minimal molecular biology expertise. The KASP genotyping method is an established PCR-based protocol, and reagent products are readily available globally [36]. The cost per sample for genotyping by MMS is up to an order of magnitude lower than that of genotyping with saturated genotyping arrays. Looking at the UK market value, for example, genotyping costs for a 25 KASP marker set are ~£4 per sample (or less if conducted in-house), compared with ~£35 per sample for the purchase and processing of a wheat or barley Axiom array. For a genebank regenerating and distributing 1000 accessions annually, the related MMS annual operational cost for molecular barcoding would be £4000, as compared with £35,000 if an array were used.

Moreover, the MMS application does not require expertise in bioinformatics or access to high-performance computing, which can be restrictive for many crop genebank teams. SNP-based genotyping using reduced marker sets provides a practical alternative to sequence-based genomic approaches [37,38,39].

In this work, we show that developing diagnostic SNP panels comprising the minimum number of markers necessary to differentiate all pairwise comparisons can improve genebank management [36]. Such marker panels can be used as a quality control (QC) during regeneration to ensure that the old seed stock sown is ‘identical’ to the fresh seed lot generated. Similarly, they can be routinely used as quality assurance (QA) for PGR users, attesting that the delivered seed is consistently ‘identical’ to those previously deposited and referenced in the genebank database. It is important to note that the term “identical” is biologically misleading: even in the absence of human error, contamination, or cross-pollination, evolution continues to occur. Several studies in cultivated wheat [40] and wild relatives [41] document non-zero mutation rates per generation. In wheat, changes in microsatellite loci were calculated at rates up to 4.97 × 10^−3^ per generation [40], implying that absolute identity cannot be assumed. In this context, as most plant scientists would appreciate, “identical” is better understood to mean “indistinguishable at a specified resolution”.

### 3.2. Minimal Marker Sets as an Affordable Molecular Labelling Approach

In this study, we demonstrate that a minimal marker set resolution is well matched to the needs and standards of genebanks. We show that this tool can generate a unique genomic fingerprint for 96 out of the 98 studied wild barley populations, resembling wild genebank accession pools [28,42]. Genebank accessions of crop wild relatives are expected to represent the internal variation within the sampled wild population [14]. Therefore, separating individuals within a wild population would represent a sub-accession resolution. Indeed, the minimal marker set was often not robust enough to separate individuals within single wild populations. We also demonstrated that the separation resolution can be efficiently increased by the simple addition of markers, thus potentially optimising the MMS total running costs according to the molecular labelling needs of any given project.

The lower average MAF and PIC values observed for wild barley (0.213, 0.218, respectively) compared to landrace barley (0.424, 0.365) and modern cultivars (0.343, 0.332) (Supplementary Table S12) are indicated by the overall decreased 24BMMS discrimination ability of the tested wild populations. It was previously shown that wild barley populations’ diversity is driven by regional and local patterns, often displaying sharp geographic differentiation over short distances [26].

Marker sets for variety identification have been developed in various species, leveraging SNP arrays and SSR markers [43,44,45]. Each crop type will require a different number of DNA markers, depending on the genetic diversity of the crop, the availability of markers, and the number of varieties to be distinguished [46]. Gao et al. [45] developed a diagnostic panel of 43 SNPs that could identify 429 common wheat varieties in China. Perry and Lee [22] selected 32 SNPs to identify Canadian hexaploid wheat varieties using the TaqMan OpenArray system and 16 SNPs to identify Canadian tetraploid wheat varieties [47]. Lee et al. [48] further expanded the panel to 32 SNP markers for identifying Canadian wheat varieties. Nguyen et al. [49] reported that as many as 192 markers were required to distinguish more than 90% of assessed pumpkin varieties, considerably more than reported in Siberian rye (52 markers) [50], rice (36 markers) [51], and soybean (23 markers) [52]. Henning et al. [53] found that only seven markers were required to differentiate the USDA-ARS hop collection. Wang et al. [54] selected a panel of 80 SSR markers that was effective in evaluating the distinctiveness of over 1000 Chinese wheat varieties.

Winfield et al. [55] designed an Axiom^®^ 35k Breeders’ array that effectively identified genetic diversity across wheat germplasm. Likewise, Singh et al. [56] applied next-generation sequencing for efficient genebank curation for *Aegilops tauschii* collections. These approaches undoubtedly provided high-resolution genotyping and powerful tools to identify duplicated accessions within and between genebanks; however, they required expensive technology and substantial expertise in large-scale data handling, making them impractical for routine genebank operations. Whole-genome resequencing of the wild barley diversity collection was done to exploit allelic variation in *Hordeum vulgare* subsp. *Spontaneum* for identifying and exploiting genetic variation for cultivated barley improvement [57].

An approach more comparable to ours was published by Tang et al. [58] who selected 48 KASP markers to classify and analyse 518 conventional and hybrid rice varieties. The panel achieved a 100% discrimination rate between 53 conventional *indica* varieties and 193 hybrid varieties and could distinguish 89.1% conventional *japonica* rice from different breeding institutes. The markers developed in this study achieved similarly high discrimination rates: 98.84% between 345 *Triticum durum* landraces collected globally during the 1920s and 1930s, 97.5% between 160 cultivars collected globally between 1962 and 1989, and 97.31% between a recently assembled global panel, using 25 markers. The strength of the MMS developed and validated in this work lies in its ability to distinguish century-old landraces from globally diverse origins, as well as modern varieties and ancestral crop wild relatives.

In barley, previous studies have shown that genotyping technologies can make variety identification more efficient and cost-effective [46,59,60]. Bayer et al. [9] used a 50K-iSelect SNP Array for barley genotyping, but relying on high-throughput SNP arrays is too costly and less accessible for routine genebank work. Smaller panels were created by Lee et al. [46] using 24 markers in the TaqMan platform to distinguish Canadian barley varieties. Owen et al. [61] employed 38 markers in the KASP genotyping platform to support the seed certification system in contemporary Scottish barley varieties.

### 3.3. Applications of Minimal Markers in Genebank Management

Genebank curators face the challenge of ensuring accurate identification of seed stocks and maintaining the genetic integrity of collections. Human errors during routine genebank operations, such as mislabelling or unintended admixtures, have been frequently reported in various plant species conserved at diverse genebanks. As such mistakes are not fully preventable, they can naturally lead to the deterioration of a genebank collections’ value and restrict future germplasm use in breeding and research. The two MMSs described in this work are operational in the GRU. Here are four examples of their use within the genebank workflows.

1. Quality control (QC) during regeneration:

For material already genotyped by the MMS, one of the seeds returning from the regeneration plot is genotyped and compared with the original registered fingerprints of the originator stock. If the tested seed fingerprint matches that of its progenitor, we consider the stock an MMS QC seed. If, however, it is different from the expected marker combination, a second seed test would confirm the mistake. In this case, if the wrong combination exists elsewhere in the regenerated field or greenhouse, the sample’s identity can be corrected and switched back. If not, the mistake can be attributed to cross-pollination or to an unknown seed origin, and the new sample is thus discarded.

If the original stock was not yet tested, seeds are morphologically compared according to the traditional genebank standard protocols. However, when an apparent discrepancy is observed between an old seed stock and its supposedly regenerated progeny, the two packets are genotyped and molecularly compared using the MMS. In a case of discrepancy, the new progeny is discarded, and the old one is marked for regeneration in the following season.

2. Quality assurance (QA) of distributed seed stocks:

Genebank-genomic projects enhance the PGR value as PGR users can apply association genetics, investigate genomic variation in known genes, or apply marker-assisted breeding based on previously published information. When seeds are ordered from genome-sequenced collections, the MMS can be used to ensure that the dispatched seed stock is ‘identical’ to the published genomic information for the accession.

3. Preventing human error in field regeneration of breeder lines or mutagenised populations:

As shown, the MMS cannot distinguish between mutants or breeder lines sharing the same genetic background. When we regenerate such populations in the field, we include control varieties randomly scattered across 15–20% of the plots. The control varieties and the population genetic background are distinct, and their MMS genomic fingerprint is known. In such a case, we cannot prevent a local mix-up (e.g., a seed mix-up or cross-pollination between two adjacent plots), but we can ensure that the field is overall highly intact by confirming the molecular identity of all the scattered control varieties.

4. For cereal wild relatives and landraces showing high intrinsic accession diversity, we stabilise a line from each population through single-seed descent (SSD) and fingerprint it. While the SSD collection has reduced diversity overall, it is more amenable to reproducible pre-breeding and genomic applications, including MMS fingerprinting QC and QA.

Together, these demonstrate how minimal marker panels provide a rapid, cost-effective, and reliable method for detecting mislabelling, contamination, or regeneration errors, thereby strengthening QC and QA procedures in genebank management and seed multiplication pipelines.

Many genebanks have limited resources to assess every accession for genetic diversity, correct origins, and species classification [62]. A recent study [63] on 600 hexaploid wheat landraces conserved at ICARDA using the DArT assay showed a significant overlap/admixture. A study on barley collections from the Ethiopian genebank also showed that there was significant admixture [64]. Previous studies conducted using a set of 93 to 235 markers on rice germplasm conserved at the AfricaRice genebank have reported the presence of admixture between *O. glaberrima* and *O. sativa* [65] and between *O. glaberrima* and *O. barthii* [66]. It is extremely difficult and often impossible to correct a seed mix-up. By applying a minimal marker identification tool before a regeneration cycle, it is possible to detect and reverse unpreventable errors at a stage where correction is still possible.

Another persistent challenge in genebank operations is the management of duplicate accessions [67]. Large ex situ collections, particularly of crops such as barley, wheat, and rice, often accumulate redundant entries due to repeated collecting missions, germplasm exchange between genebanks, inconsistent or incomplete passport data, and synonymy in naming. The FAO’s Second Report on the State of the World’s Plant Genetic Resources for Food and Agriculture states that although approximately 7.4 million accessions are maintained in genebanks worldwide, only 25–30% (1.9–2.2 million) are distinct; the rest are likely duplicates across collections [68]. Singh et al. [56] demonstrated that over 50% of *Aegilops tauschii* accessions were duplicated across genebank collections, largely due to sharing, re-numbering, and incomplete documentation. Analysis of the rice germplasm revealed that about 28.7% of 223,397 accessions were similar to one or more other accessions, grouping into numerous similarity clusters, indicative of duplication due to shared origins or naming inconsistencies [69]. It has been reported that around 50% of global ex situ germplasm is concentrated in just 10 crop species, with wheat, rice, and barley among the top contributors, underscoring the significant potential for duplicate entries in such large collections [70]. These duplications inflate collection sizes without adding new genetic diversity, creating unnecessary burdens on conservation costs [24].

In this context, it is important to consider the boundaries and limitations of MMS fingerprinting. While two identical fingerprinted lines are likely duplicates, they might also be lines originating from the same pedigree or sharing the same ancestral wild population; thus, it is not unlikely that both ‘identical’ lines are worth conserving. However, in an unfortunate situation, when ex situ collection size must be rationalised, prioritising based on MMS fingerprints is a safe method to minimise diversity loss.

We demonstrated that the described MMS resolution limits its use for breeder lines, mapping populations, and unstable wild populations, but is ideal for stable cereal varieties or lines derived from landrace and wild populations and stabilised through SSD. In this study, we demonstrate the minimal marker algorithm use for fingerprinting crops’ gene pools with a strong tendency toward inbreeding. Previous work on apple trees demonstrated their reliable use for fingerprinting fruit trees [69] that are highly heterozygous but clonally propagated, thus stable in the horticultural context. The method’s limitations and advantages should therefore be considered for each crop group separately.

### 3.4. Beyond Genebanking

In addition to cost-effective germplasm fingerprinting for which the MMSs were developed and optimised, MMS genotyped collections could also benefit from a better understanding of the conserved germplasm diversity. The MMS can be used to generate principal component analysis (PCA) and phylogenetic trees, as exemplified in Supplementary Figures S1 and S2, thereby gaining insights into the relatedness of germplasm subgroups. The PCAs separated the fingerprinted accessions by germplasm background classification, and this grouping was consistent with the phylogenetic relationships observed in the Neighbour-Joining trees. These analyses exemplify the potential of the developed MMS for germplasm diversity studies and germplasm panel selection.

Minimal marker application for crop identification can play an important role beyond the genebank management scope that was emphasised in this study. The broader implications of this work extend into breeding and crop research, where molecular barcoding can streamline genotype selection and tracking. The ability to uniquely identify and track accessions over time enhances the integrity of long-term genetic resource conservation efforts. Variety registration in many parts of the world is based on tests to establish that every new variety is Distinct, Uniform, and Stable (DUS test) [71]. The tests for distinctness could clearly benefit from an agreed set of markers, rather than an agreed set of morphological changes.

Inexpensive genetic identification can also support tighter links between crop genebanks and farmers. For example, it can facilitate screening of broad genetic diversity using bulked accession mixes. If the accessions within the mix are fully separable by a dedicated MMS, any plant of interest in the farmer’s field, as well as its progeny seed, can be fingerprinted and identified for many downstream applications in crop improvement.

## 4. Materials and Methods

### 4.1. Plant Materials and Availability

All the germplasms and their current genebank identity codes and descriptors are listed in the Supplementary Tables: two barley panels, *Hordeum vulgare* and *Hordeum spontaneum,* in Supplementary Tables S3 and S6, respectively, and the tetraploid wheat collections in Supplementary Tables S9–S11.

The PGR panels in this study were selected to encompass the broadest possible genetic diversity. They were designed to include all PGR categories representing a crop gene pool: landraces, modern crop cultivars, wild populations, breeder lines, and mapping populations, as indicated in the text.

For barley, the study included 1147 modern cultivars, landraces, and wild accessions. The domesticated accessions (*Hordeum vulgare*) are listed in the BBSRC Small-Grain Cereal Collection and available at https://www.seedstor.ac.uk/. The wild barley accessions (*Hordeum spontaneum*), collected in 1977 jointly by the UK Agricultural Research Council and the Hebrew University of Jerusalem, are available from the corresponding author by email.

For tetraploid wheat collections, we used a total of 888 accessions: 505 from the Germplasm Resources Unit (GRU) at the John Innes Centre (JIC), UK (available at https://www.seedstor.ac.uk/), and 383 imported global tetraploid collections available from ICARDA, the AGG, and USDA as listed in the corresponding Supplementary Tables.

### 4.2. DNA Extraction

Genomic DNA was extracted from 14-day-old leaf tissues using the JIC genotyping service protocol (DNA_extraction_protocol.pdf), adapted from MA Pallotta et al. [72].

### 4.3. SNP Selection and Primer Design

SNP selection was performed using a Perl script developed by Winfield et al. [73] available on https://github.com/pr0kary0te/minimalmarkers/blob/f67eb6a57b05c4ce52e024ad406f3ed2bb142f33/select_minimal_markers.pl#L1-L270 (accessed on 12 April 2024). The script was applied independently to the durum and barley datasets. All the 345 durum wheat collections were genotyped using the 35K Axiom Wheat Breeders’ Genotyping Array from Affymetrix at the University of Bristol Genomics Facility, Bristol, UK. Supplementary Figure S3 describes the population structure analysis (K = 3). We ran the minimal SNP finding analysis on the durum accessions using 35K Axiom breeders’ array data (available at https://www.seedstor.ac.uk). This resulted in 21 markers that discriminated all 345 accessions (Supplementary Table S1). As the UK wheat community is transitioning to a new TaNGv1.1 Axiom array [29], we repeated the analysis using the 7144 SNPs shared between the 35K breeders’ array and TaNGv1.1. This analysis produced a set of 25 markers that discriminated all durum accessions (Supplementary Table S4). For the barley datasets, we used an existing published genebank-genomic resource that describes the global crop population structure, comprising 171,263 SNPs across 22,626 global barley accessions conserved at the Leibniz Institute of Plant Genetics and Crop Plant Research (IPK) [15].

The minimal marker script, as fully described in [73], first identifies the SNP marker with the highest MAF score. This marker splits the sample into two pools. After this, the script evaluates all other markers to see which can differentiate the highest number of samples that were not split by the first marker, creating two pools of samples. The script iterates this process with a logarithmic division of samples until adding more SNPs provides no further splits or until all samples are resolved. The final marker set is considered haplotype-optimised [29]. Importantly, in each iteration, only previously unresolved accession pairs are considered. For each remaining SNP, the algorithm computes incremental discrimination, defined as the number of accession pairs newly distinguished by that SNP but not by any previously selected SNP. The SNP providing the greatest incremental discrimination is selected at each step. By this process, any markers in LD with a previously selected marker cannot add value for sample differentiation and are therefore not selected by the algorithm. To optimise genomic coverage and enhance separation power, additional markers were manually selected from the script-generated list based on their differentiation scores. The low LD levels among all markers were then confirmed by estimating pairwise linkage disequilibrium (LD) as squared correlation coefficients (r^2^) between markers, visualised using heatmaps (Supplementary Figure S4).

### 4.4. Development of Primers

For each SNP marker, two allele-specific forward primers and one reverse primer were designed (Supplementary Table S4). A ~50 bp upstream sequence flanking the SNP site was used to ensure primer specificity. To minimise amplification issues, primers were designed to avoid repetitive sequences and ensure optimal GC content. Primers carrying standard FAM tail 5′-GAAGGTGACCAAGTTCATGCT-3′ and VIC tail 5′-GAAGGTCGGAGTCAACGGATT-3′ with the polymorphic base (targeted SNP) positioned at the 3′ end were synthesised by Sigma-Aldrich, Gillingham, UK (https://www.sigmaaldrich.com/gb/en/quick-order (accessed on 12 April 2026)).

### 4.5. Primer Optimisation and SNP Replacement

Ineffective or unreliable primers were either redesigned or replaced following a structured protocol; the reverse primer was relocated to a more suitable genomic region. If no suitable reverse primer location was available, the forward primers were switched to the opposite DNA strand, and a new reverse primer was selected. If neither adjustment yielded effective primers, the SNP marker itself was replaced using a modified SNP selection script that disregarded the ineffective primer and allowed for the forced inclusion of known markers available on https://github.com/pr0kary0te/GenomeWideSNP-development (accessed on 15 May 2024). This stepwise approach ensured the highest accuracy and efficiency of the final minimal marker set, enhancing its utility for genebank management and genetic resource conservation.

### 4.6. Genotyping Protocol

In a 384-well plate, 1.8 µL of diluted DNA (5–10 ng/µL) was dispensed into each well and dried in an incubator. A mastermix of 580 µL sterile DiH20, 580 µL PACE mix (3CR bioscience), and 17 µL of the primer mix was prepared, after which 2.4 µL of the mixture was dispensed into each well using a Meridian liquid-handling system. The primer mix consisted of 46 µL sterile DiH20, 12 µL forward primer 1, 12 µL forward primer 2, and 30 µL common/reverse primer. Amplification took place using an LGC hydrocycler and occurred under the following conditions: 94 °C for 15 min, then 10 cycles of 94 °C for 20 s, 65–57 °C for 60 s (dropping 0.8 °C per cycle), followed by 38 cycles of 94 °C for 20 s, 57 °C for 60 s. Once the amplification programwas complete, the plates were centrifuged for less than 30 s, first topside down and then topside up. Fluorescence was then detected at room temperature using a PHERAstar microplate reader (BMGLABTECH, Offenburg, Germany). Genotype calling was scored using the LGC Biosearch Technologies Kraken™ LIMS; KlusterCaller™ software (v2.23.0.7) (A negative (no DNA) and a positive (wheat paragon CV) control were included for the MMS optimisation experiments. Following the MMS optimisation, each genebank accession was evaluated once. The overall non-calling rate (including any marker ambiguity call) was 2.8% for barley and 5.1% for durum wheat across all the reported wet lab experiments. Non-calling was considered as missing data.

### 4.7. Data Analysis, Visualisation and Statistics

GenAIEx v. 6.5 [74] software was used for multilocus analysis. The markers’ distribution throughout the durum wheat and barley genome was graphed using the R version 4.4.2 package chromoMap, svglite, and ggplot2. The Leaflet R package was used for the wild barley collection sites mapping.

To infer the optimal subpopulations that existed in the 345-durum landrace training population, a Bayesian model-based clustering approach was implemented using STRUCTURE v.2.3. The best K value was estimated as Delta K (ΔK) from Structure Harvester. The estimated ∆K suggests the presence of three genetic subgroups in the collection panel (Supplementary Figure S3).

The KASP Markers’ statistics are summarised in Supplementary Tables S12 and S13 for barley and durum wheat, respectively, including minor allele frequency (MAF), polymorphic information content (PIC), expected heterozygosity (He), and observed heterozygosity (Ho), which were calculated using custom R scripts. MAF was defined as the frequency of the less common allele, He as $1-(p^{2}+q^{2})$, Ho as the proportion of heterozygous individuals, and PIC for biallelic markers as $1-(p^{2}+q^{2})-2p^{2}q^{2}$, where p and q represent allele frequencies.

Principal component analysis (PCA) was performed using the prcomp function. Genetic relationships among accessions were inferred using a Neighbour-Joining phylogenetic tree constructed from Euclidean genetic distances with the ape package. To assess the robustness of the minimal marker set (MMS), marker failure scenarios were simulated. Random subsets of markers (ranging from 1 to n − 1) were removed, and for each level, 200 independent simulations were performed. The discrimination rate was calculated as the proportion of unique multilocus genotypes among all accessions.

To assess the robustness of the minimal marker set (MMS), marker failure scenarios were simulated. Random subsets of markers (ranging from 1 to n − 1) were removed, and for each level, 200 independent simulations were performed. The discrimination rate was calculated as the proportion of unique multilocus genotypes among all accessions. All data processing and visualisation were performed in R using the packages ggplot2, ape and ggrepel.

## 5. Conclusions

It can be envisaged that, in the future, all ex situ conserved crop collections will be genome-sequenced at a high resolution, and that data analysis and the transfer of such genomic resources will be feasible for global genebanking. However, it may take decades for most collections to reach this point. In the interim, safeguarding the vast collections of existing germplasm requires practical, cost-effective molecular solutions that maintain genetic identification and prevent dissociation of germplasm from associated data. The minimal marker solution presented here can serve as an interim, low-cost, rapid, and scalable method for identification. Extending the use of SNP-based genetic identification would enhance genebanking cost-effectiveness, supporting global food security.

This study demonstrates the successful development and application of minimal marker sets for barley and durum wheat in existing genebank collections. Unlike many previous varietal identification panels, optimised for specific breeding programmes or geographic subsets, these marker sets have been tested across diverse collections, including wild relatives, landraces, and historical and modern germplasm cultivars. The consistently high discrimination power demonstrates that when carefully selected, a small marker panel is sufficient to fingerprint a wide selection of accessions.

The reliable performance of these markers confirms their utility as a practical tool in genebank management. These marker sets provide identification of unpreventable human errors during the regeneration of large collections (e.g., seed mix-ups, field mislabelling, mistakes during sowing or harvesting) at a stage when the mistake can be rectified before it compromises the value of stored germplasm.

## Figures and Tables

**Table 1:** Summary of the separation power results of the minimal markers over different tetraploid wheat and barley panels and experiments. For barley, the multiple landrace rows represent sequential validation steps using different marker set sizes and increasing landrace sample sizes, rather than repeated analyses of a single homogeneous landrace group.

Crops	Training Population (Size) for in Silico Marker Selection	Analysis Population Size (Wet Lab KASP)	Germplasm Source	Number of Markers	Discrimination %
Tetraploid Wheat	The Watkins Durum Landrace collection (345)	345	The Watkins Durum Landrace Collections	25	98.84
		160	BBSRC Small-Grain Cereal Collections	25	97.5
		260	Global Durum Wheat Panel (GDP)	25	97.31
		122	Global Tetraploid Wheat Wild Relatives	25	89.44
Barley	Global Barley Collection (500)	96	BBSRC Small-Grain Cereal Collections	12	93.75
		96	BBSRC Small-Grain Cereal Collections	24	97.92
		384	Landraces (Set 1)	12	70.57
		384	Landraces (Set 1)	24	90.36
		284	Landraces (Set 2)	24	96.12
		668	Merged Landraces Experiments	24	92.22
		95	Wild Barley (Set 1)	24	97.89
		182	Wild Barley (Set 2)	24	94.79
		287	Merged Wild Barley Experiments	24	95.12

## Data Availability

The original contributions presented in this study are included in the article/Supplementary Material. Further inquiries can be directed to the corresponding author.

## Supplementary Materials

The following supporting information can be downloaded at: https://www.mdpi.com/article/10.3390/plants15081219/s1. Figure S1: Minimal marker-based principal component analysis and phylogenetic tree of the studied barley accessions. Figure S2: Minimal marker-based principal component analysis and phylogenetic tree of the studied durum wheat accessions. Figure S3: Population structure analysis for the Watkins durum wheat training population. Figure S4: Pairwise linkage disequilibrium (LD) analysis of the Minimal marker set. Table S1: The 345 durum wheat Watkins landrace (Training population) accessions’ names discriminated by 21 named KASP markers. Table S2: The 500 randomly selected accessions of barley (IPK Training population) discriminated by 12 KASP markers. Table S3: The barley accessions used in this study for wet-lab minimal marker set validation. Table S4: A list of markers developed in this study; marker ID and their corresponding primers. Table S5: A list of non-discriminated barley lines and related passport data. Table S6: A List of Wild barley accessions (*Hordeum spontaneum*), collection site GPS coordinates, and the minimal marker set genotyping results. Table S7: Minimal marker set separation results within wild barley populations. Table S8: Minimal marker set separation results within barley breeder line populations. Table S9: Durum wheat accessions used in this study; passport data and minimal marker set genotyping results (germplasm available from the JIC GRU, UK). Table S10: Durum wheat accessions used in this study, along with their study; passport data and minimal marker set genotyping results (available from AGG, ICARDA and USDA). Table S11: List of tetraploid wheat wild relatives with their corresponding subspecies sourced from the AGG and USDA genebanks and their minimal marker set genotyping results. Table S12: The statistical evaluation of the minimal marker set wet-lab performance: PIC, MAF, Ho, and He of markers in landraces, modern cultivars and wild barley germplasm groups. Table S13: The statistical evaluation of the minimal marker set wet-lab performance: PIC, MAF, Ho, and He of markers in durum landraces, GDP imported cultivars, and wild tetraploid germplasms.
