# Supplementary material for: Molecular Labelling Tool for Cereal Genetic Resources Management Derived from Barley and Tetraploid Wheat Genebank-Genomics Projects
Source: Plants (Basel). 2026 Apr 16;15(8):1219. doi: 10.3390/plants15081219 (PMC13119740; doi:10.3390/plants15081219)
Supplement: Supplementary file 1 [file plants-15-01219-s001.zip › Supplementary figures WZ-V3_03042026.pdf]

Molecular Labelling Tool for Cereal Genetic Resources Management Derived from Barley and Tetraploid Wheat Genebank-Genomics Projects

Workie Zegeye [1,2], Amanda Burridge [1,3], Ajay Siluveru [1], Simon Orford [1], Liz Sayers [1], Richard Gorham [1], Richard Horler [1], Gary Barker [3] and Noam Chayut [1,*]

[1] Germplasm Resources Unit, John Innes Centre, Norwich Research Park, Colney Lane, Norwich, NR4 7UH, United Kingdom.

[2] Department of Agricultural Biotechnology, University of Gondar, Ethiopia.

[3] University of Bristol, 24 Tyndall Avenue, Bristol BS8 1TQ, United Kingdom.

* Correspondence: Noam.Chayut@jic.ac.uk; +44-1603-450000

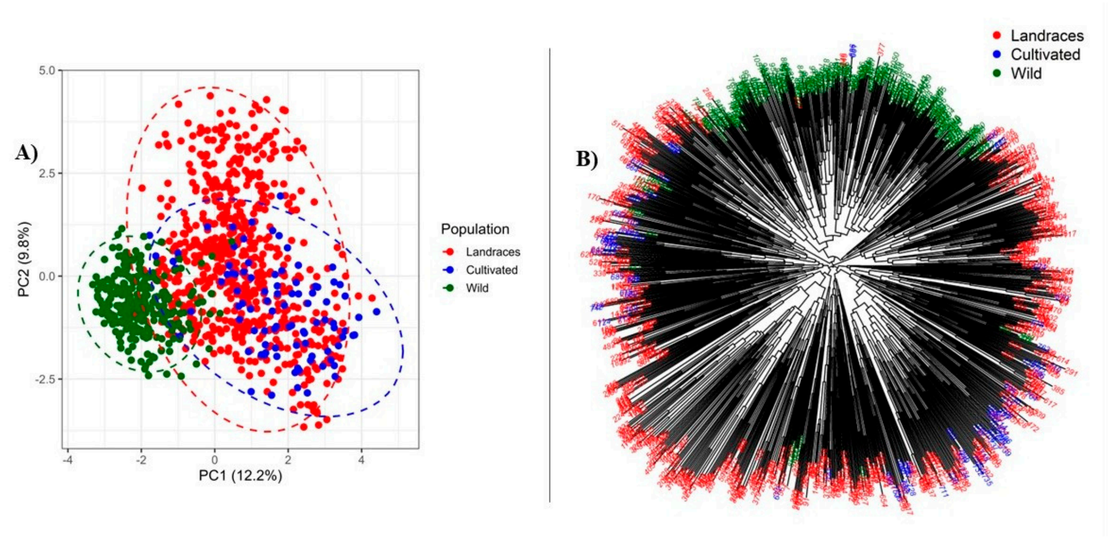

Figure S1. Principal Component Analysis (A) and phylogenetic tree (B) of the studied barley accessions, based on genotyping data from the 24 markers comprising the newly developed minimal marker set.

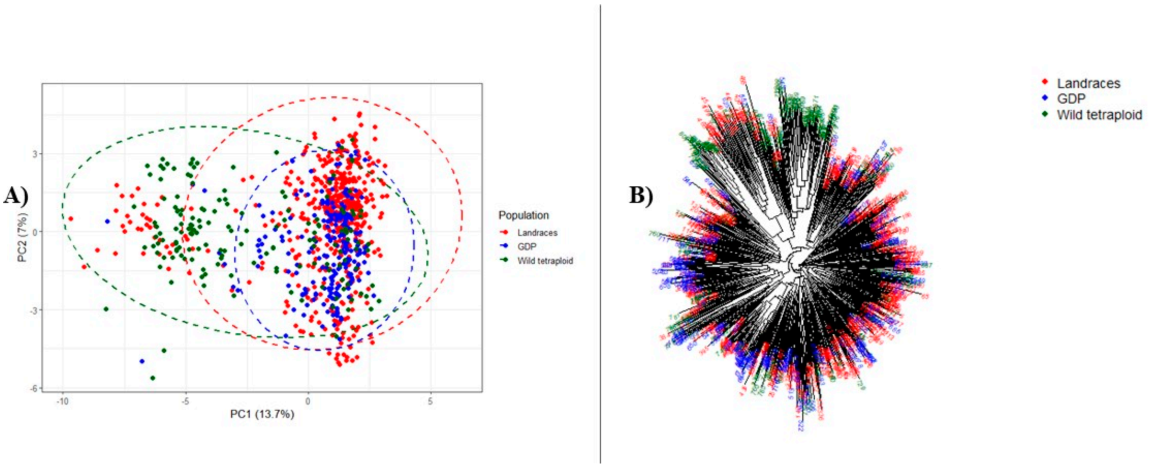

Figure S2. Principal Component Analysis (A) and phylogenetic tree (B) of the studied Tetraploid wheat accessions, based on genotyping data from the 25 markers comprising the newly developed minimal marker set.

A)

K	Reps	Mean LnP(K)	Stdev LnP(K)	Ln'(K)	|Ln''(K)|	Delta K
1	3	-1422731.066667	169.787642	—	—	—
2	3	-7659518.500000	10797453.032272	-6236787.433333	12399879.800000	1.148408
3	3	-1496426.133333	94857.103527	6163092.366667	14900110.800000	157.079546
4	3	-10233444.566667	4359602.517263	-8737018.433333	39050625.233333	8.957382
5	3	-58021088.233333	62663959.547461	-47787643.666667	178288059.300000	2.845145
6	3	-284096791.200000	246865983.656487	-226075702.966667	26690882.000000	0.108119
7	3	-483481612.166667	248893223.622619	-199384820.966667	410098132.033333	1.647687
8	3	-1092964565.166667	173407593.768444	-609482953.000000	78464199.533334	0.452484
9	3	-1623983318.633333	517372678.609674	-531018753.466666	259601847.433333	0.501770
10	3	-1895400224.666667	767709996.755763	-271416906.033334	—	—

B)

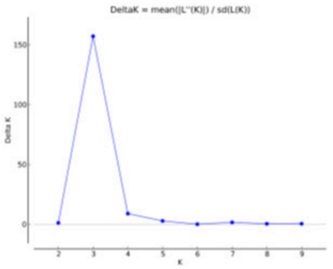

C)

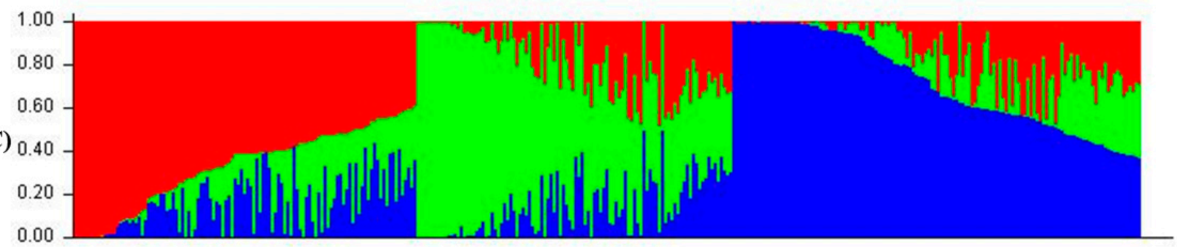

Figure S3. Population structure analysis for the Watkins durum wheat training population. **(A)** and **(B)** The best K value estimated as Delta K (ΔK) from Structure Harvester. **(C)** a Bayesian model-based clustering approach to estimate the optimal subpopulations and the membership probability of each genotype to the subpopulations using STRUCTURE v.2.3 software. The estimated ΔK suggests the presence of three genetic subgroups in the panel.

A)

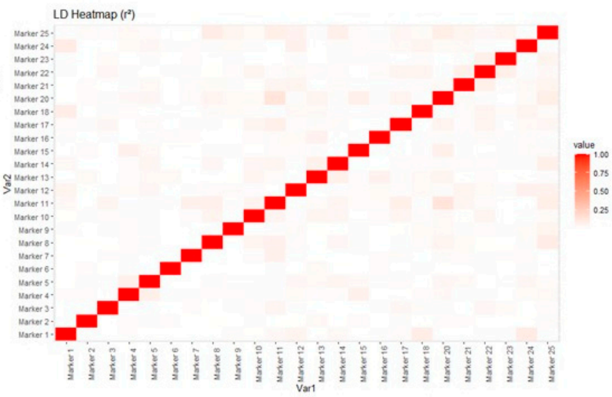

B)

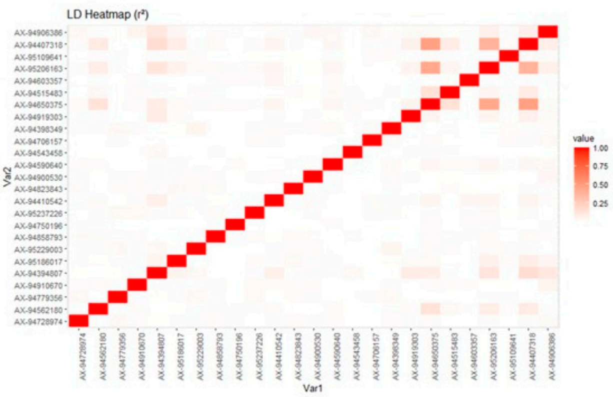

Figure S4. Pairwise linkage disequilibrium (LD) analysis. **(A)** LD observed among barley markers, and **(B)** LD observed among durum wheat markers.
